# Mining Big Neuron Morphological Data

**DOI:** 10.1155/2018/8234734

**Published:** 2018-06-24

**Authors:** Maryamossadat Aghili, Ruogu Fang

**Affiliations:** School of Computing and Information Sciences, Florida International University, Miami, FL 33174, USA

## Abstract

The advent of automatic tracing and reconstruction technology has led to a surge in the number of neurons 3D reconstruction data and consequently the neuromorphology research. However, the lack of machine-driven annotation schema to automatically detect the types of the neurons based on their morphology still hinders the development of this branch of science. Neuromorphology is important because of the interplay between the shape and functionality of neurons and the far-reaching impact on the diagnostics and therapeutics in neurological disorders. This survey paper provides a comprehensive research in the field of automatic neurons classification and presents the existing challenges, methods, tools, and future directions for automatic neuromorphology analytics. We summarize the major automatic techniques applicable in the field and propose a systematic data processing pipeline for automatic neuron classification, covering data capturing, preprocessing, analyzing, classification, and retrieval. Various techniques and algorithms in machine learning are illustrated and compared to the same dataset to facilitate ongoing research in the field.

## 1. Introduction

### 1.1. Motivation

Neurons are the building blocks of the nervous system. They exchange information to control the entire body. Therefore, deciphering the complex functions of neurons is fundamental to our ultimate understanding of memorization, logical thinking, and learning abilities. It is reported that there are 86 billion neurons in the human brain [1]. Every neuron is composed of three basic parts: the dendrite, the cell body, and the axon. But they vary in the number of dendrite branches, size, and shape. These variations lead to the different functionalities of particular neuron types. All neurons belong to at least one of the three basic types: (1) sensory neurons which receive external stimuli and convert them to internal impulses that are transmitted to the brain, (2) interneurons that convey these signals between neurons, and (3) motor neurons that pass the signals from the brain to different organs. Despite this general classification, there are neurons which have not been well-defined in neuroscience [2].

Neuroscientists have verified that there is a relationship between the form and structure of neurons, their functionality, and underlying connectivity [3–5]. Neuromorphology is a multidisciplinary research field which involves various scientific domains including biology, chemistry, computer science, and machine learning. This field studies the neural system's form, function, connectivity, physiology, and molecular properties [5–7]. They have also corroborated that neurons' morphology differs based on the different species, regions in the living body, cell functions, and developmental stages [6]. Despite the extensive research in this field, a general agreement about all the neuron types has not yet been reached.

The convoluted shape of neurons, coupled with their subtle structural differences between types, exposes a new challenge for researchers in recent years. The advent of new technologies such as bright field microscopy, confocal 2-photon microscopy, and automatic and semiautomatic neuron tracing has facilitated and accelerated the process of 3D neurons images reconstruction and it has allowed the number of neural images to grow exponentially. Therefore, to understand and explore this complicated data, it is necessary to automate the neuron classification process to keep up with the increasing amount of accumulated data.

Most of the efforts in the past decades mainly have depended on human endeavors to manually classify the neurons. However, in recent years neuroscientists have started using artificial intelligence and machine learning techniques to automatically subdivide the neuromorphological space [8–15].

### 1.2. Transition to Automated Neuron Classification

Due to the importance of the neuromorphology along with the burdensome task of manual classification, different lines of research have evolved, which exploit computational approaches for automatic neuron classification. The importance of the neuron morphology, the laboriousness, and the considerable cost of the current manual process leads to the development of a repository named Neuromorpho.org. This is a public dataset, lumping together many of the available neurons' morphology research data along with the corresponding publications. It has been established online to provide an easy access platform for sharing the valuable results of labor intensive research on neurons from various sources. This database is growing steadily to become a complete reference of neuronal morphology studies. It is composed of tens of thousands of 3D cell images with the corresponding metadata and related papers from 140 laboratories worldwide [24]. The full procedure of the dataset establishment has been provided in [16, 25].

### 1.3. Challenges of Automated Neuron Classification

Although the emergence of the Neuromorpho.org is promising for further breakthroughs in the field, the disparate sources of data, different experimental conditions, divers levels of reconstruction completeness, and lack of metadata information lead to a discrepancy in the results. However, a new course of action has started to address these issues by providing adequate standards for reporting metadata and details of digital reconstruction. This research initiative has prompted to refine and complete the metadata information in the Neuromorpho website [26, 27]. Additionally, a consistent terminology for effective data sharing and communication has been established to unify the experiments' results [28]. Due to the lack of consistent terminology for data sharing and effective communication standard, Neuroscience Information Framework has recently assembled a comprehensive lexicon to cover the neuroscience domain and proposed a unified terminology [28, 29].

As explained in the following section, the neurons' image acquisition and reconstruction process are not only prolonged but also vulnerable to human bias and judgment [29]. Therefore defining a stringent guideline and sharing the acquired data with enough comprehensive metadata will significantly help this line of research. In addition, a part of the metadata sometimes acts as a confounding variable that should be taken into account in analyzing information from different sources.

The 3D nature of the neuron's image also hampers the application of many popular methods and techniques of pattern recognition, image detection, and classification. For instance, deep learning which is an emerging field of research can be exploited normally for 2D images but some challenges should be addressed to effectively apply it on 3D images.

### 1.4. Contribution and Organization of the Paper

This survey paper provides an extensive organized overview of computational methods in neuromorphology. Most of the papers on neuromorphology are written by neuroscientists and lack a comprehensive explanation of data processing steps; all are filled with technical expressions and definitions from that field. However Vasques et al. recently reviewed most of the morphological classification research and they have briefly provided a review on methods, materials, and machine learning algorithms in neuromorphology [30]. In contrast, this survey approaches the neuromorphology from a new point of view with a broader spectrum and attempts to provide a user-friendly review for scientists in different fields to understand the type of ongoing research, opportunities, and challenges in the field. It explains the entire process of neurons classification from scratch and elaborates the way of image retrieval. A comprehensive pipeline which precisely presents the steps of neurons classification from capturing raw data to defining the final neurons' type is provided in Section 2.  Section 3 explains state-of-the-art neurons retrieval algorithms.  Section 4 presents and compares computational results and the last chapter provides conclusions, discussion, and future directions.

## 2. Neuron's Mining Pipeline

**Figure 1:**
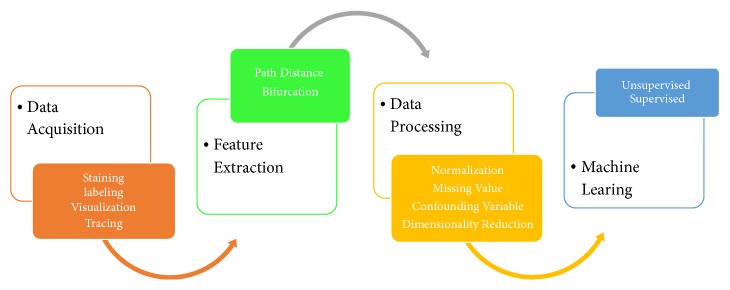
Neuron's mining pipeline.

In this section, a pipeline for the neuron mining is proposed. The steps are shown in Figure 1. Every step will be explained in depth as follows.

### 2.1. Data Acquisition

The advent of Golgi's staining technique in the late 19th century revolutionized the understanding of the brain. This technique uses light microscopy to envision neuronal tissues. Since then a number of new and promising methods have been invented which helps scientists to understand brain functions.

Constructing a well-defined 3D image of a neuron is a time-consuming and labor intensive process. Neuron staining and labeling, as the first steps of this process, can be conducted via different methods depending on the experiment design and preparation forms. Immunolabeling of cellular proteins, bulk extracellular loading, tracer injection, and genetic labeling which mark neurons intrinsically and intracellular are the most well-known techniques of staining [31].

Visualization, as the next step, is carried out via optical techniques to acquire high-resolution neuronal images. Bright field microscopy and confocal 2-photon microscopy are the most popular visualization techniques which are used to prepare the neurons' images for tracing.

Due to the neurons' complex morphology and convoluted cell preparation process, captured images have some degree of noise, corruption, and obscurity. Tracing, which is an intensive process of reconstructing the digitized image, has evolved during the years to address the aforementioned problems. In the past, it has been performed by hand and camera lucida but nowadays it is mostly done semiautomatically. However, the tracing results are still incomplete because of the imperfect staining, tissue sectioning, and low image resolution. Many research groups are currently working on the visualization and reconstruction techniques to provide higher quality 3D images via automatic methods but human intervention is still an inseparable part of the process [21, 32]. A number of different types of popular tracing software and tools are provided in Table 1. A comprehensive detail on the visualization techniques and tools can be found in [17, 33, 34].

### 2.2. Feature Extraction

**Figure 2:**
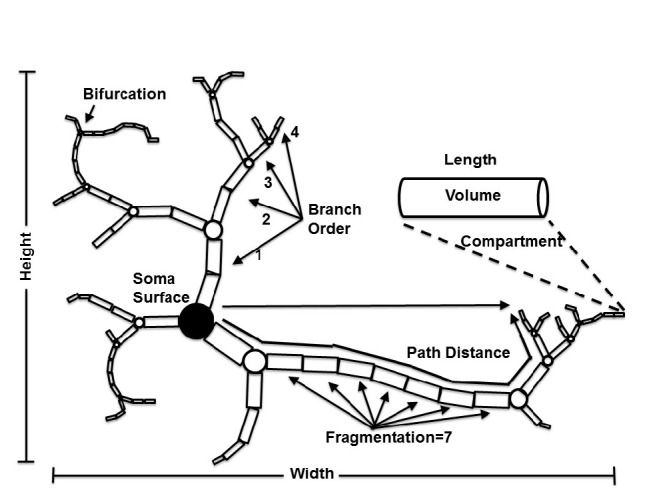
Neuron's features.

For processing and quantitative analysis of reconstructed images, neurons' features should be extracted. L-measure is one of the recent types of software that executes the morphometric calculation. This is free software and is designed to calculate more than 30 morphometric features from a neuronal reconstructed file in a wide range of formats [35]. There are some other types of alternative software for feature extraction such as Cvapp [36], Neurolucida Explorer [37], NeurphologyJ [38], and NeuronLand which can be used based on the need [39]. Some important neural features which can be extracted by L-measure are shown in the Figure 2 [40]. A detailed description of the features has been represented in [35].

### 2.3. Data Preprocessing

Since real-world data tends to be noisy, incomplete, and inconsistent, data preprocessing is necessary prior to further analysis. To achieve reliable results in the quantitative analysis, some validation, curation, and standardization steps should be performed which are considered as preprocessing. Preprocessing is specifically essential when the dataset is an amalgamation from different research laboratories. Different preprocessing steps are briefly explained below.

#### 2.3.1. Normalization

Data preprocessing is a fundamental building block of data mining. The core of preprocessing is standardization and statistical adjustment of the data. Normalization can considerably improve the classification accuracy of a number of the models. The data are not always expressed in similar units and magnitude, so data pretreatment considering certain criteria is essential prior to data analysis [41]. One of the popular methods of standardization is z-score scaling, which involves subtracting the mean from all values and dividing by the standard deviation [42–46].

#### 2.3.2. Missing Value Treatment

Missing values occur when there is no data value for some variables or features in the dataset. It is common and also a complicated phenomenon that should be addressed with an appropriate approach prior to the classification or clustering as many algorithms are unable of handling data with missing values. Most of the statistical packages ignore incomplete samples. However, invalid statistical results may be achieved as a consequence of the elimination of critical information. This method is called Listwise or Case Deletion.

A similar approach to Listwise is Pairwise deletion, which deletes or keeps the data point based on the pair scores and the application of the features in the calculation. For instance, if a sample has the value of x1,x3,x4 and y features and it misses the value of x2 and x5, the sample point is kept and when the pairs of x1 and y are needed in a calculation but if y and x5 or y and x2 are needed this sample point is discarded.

Replacing the missing value simply by zero or with attribute mean value is another way of handling unavailable values. It can be more precise if instead of attribute mean, the missing value is replaced by the mean of all samples belonging to the same class. However, this method can be misleading when the variable has large variance [47].

A more scientific approach for addressing missing values suggested the following procedure: calculating the missing value percentage and if it is less than 5% of the whole values, it can be neglected; otherwise two tests of MAR (missing at random) and MCAR (missing completely at random) should be performed to give enough confidence about whether the missing data occurred randomly or if it happened based on some corresponding situation that makes those data gathering hard. If this test becomes false, it is clear that missing data happens based on some specific situation and is systematic. Therefore using an inference method to calculate the missing values can be helpful otherwise unavailable values can not be predicted easily so Listwise Deletion or Mean Substitution can be applied based on the condition [48–50].

A better way for prediction of less biased data is applying the data mining algorithm for probable value prediction. Regression, Bayesian inference, decision trees, and clustering algorithms can be used for inferring missing values.

Regressing the missing variable based on the other independent variable is a simple solution. Regression model works well for imputation of the missing value when there is a strong relationship between missing variables and other independent variables.

Single and multiple imputations using expectation maximization are modern techniques of missing value completion. Expectation maximization, which is a kind of maximum likelihood approach, iteratively imputes the missing value based on the relationship among whole sets of variables. It adds some degree of random error to reflect uncertainty to the imputation. The algorithm will stop when the imputed variable is stabilized [51]. The propensity score method, regression modeling, and a collection of techniques called Markov chain Monte Carlo are used for data imputation. A group of well-established imputation methods such as Matrix Factorization, Singular-Value Decomposition (SVD), and K-Nearest Neighbor (KNN) have been implemented in statistical and analytical software packages such as MVA in R and Fancyimpute in Python.

#### 2.3.3. Data Unification and Consolidation

As mentioned earlier, the Neuromorpho dataset is an amalgamation of data from neurons research labs. Therefore it is not surprising that there are discrepancies in the naming of the same value in the dataset. For example, neonate developmental stages are referred by different names like “embryonic”, “infant”, “neonatal”, and “fetal” in different datasets. It is required to consolidate this data using common nomenclature prior to processing.

#### 2.3.4. Address Imbalanced Dataset

Imbalanced classes are those that have majority values in one type (more than 90%) and the remaining in the others. Accuracy is not an appropriate metrics for the classification performance in these datasets. In order to have a correct understanding of the classification performance, it is recommended to calculate F-measure, precision, and recall in the future research. Oversampling and undersampling also are popular techniques that sometimes are used to address imbalanced data [52].

#### 2.3.5. Exclusion of Confounding Variable

While experimental condition, staining model, imaging resolution, and all other empirical details dramatically affect some of the parameter calculations, feature selection should not be done blindly. Polavaram et al. claimed that axonal morphologic features and branch diameter are extremely dependent on the experimental conditions so they excluded them from the pool of features prior to analysis [46]. Different preparation mechanisms, shrinkage during tissue processing, and slicing artifacts all impact the neuron's images. Scrupulous attention to these details improves the classification accuracy.

#### 2.3.6. Dimensionality Reduction

Reducing the feature space by obtaining a set of uncorrelated variables is a popular technique in big data analysis. The remaining features should be selected carefully to exhibit the original data variability. Reduction of the attribute space to enhance the classification performance and reduce the process time is the main goal of this technique. When the dimension is reducible to two, data visualization is possible which provides a better understanding of data distribution [53, 54].

Principle Component Analysis (PCA) is the most well-known technique of dimensionality reduction in neuroscience studies. This method transforms a large set of features to a smaller set in a way that remaining set represents the original variance in the data. In other words, PCA identifies the most dominant features among the entire set and solve the curse of dimensionality which improves the classification accuracy [55].

Costa et al. applied PCA and canonical analysis on a massive dataset of 6000 neurons from Neuromorpho database to decrease the 20 extracted features to only two, which explained most of the data variability. Their result shows that cells with similar types, region, and species tend to form a cluster together and also these clusters become more substantial after applying PCA. But there is not enough evidence to answer comparative questions such as “whether the neurons of interrelated species have same morphological traits because of same habits or coexistence” or “how a neurons' morphology evolved in a species” [40].

Polavaram et al. used L-measure software to extract more than 100 features from the data which is captured from Neuromorpho.org. They applied PCA to identify the most important morphological parameters which help to perform structural classification. Their results corroborate that while direct assessment of large-scale heterogeneous dataset can not uncover meaningful patterns, by applying PCA as an effective feature space reduction, capturing the relationship between metadata and clusters become feasible. They also reported that among specific cell types and animal species there are some morphological differences that are not sensitive to the origin laboratory [46]. There are multiple pieces of research which have adopted PCA in different datasets in a similar style [56, 57].

In contrast to aforementioned studies, one study was not able to find meaningful improvement in the classification by applying PCA. In this experiment 67 morphological features of mouse frontal, visual, and somatosensory cortex were measured and cluster analysis was performed two times, one time, without applying PCA and another time after applying it, to compare the dimensionality reduction effect in the data. This experiment displays no obvious difference between those two runs rather than some cells rearrangement [44]. It suggests that while PCA has rendered promising results in several bioscience studies it is not practical [40, 46, 56–58].

PCA attempts to reduce the reconstruction error by the best transformation, so in the data with nonlinear dependencies, it can not consider higher order relations. Furthermore, PCA compresses the attributes and makes a new combined attribute; thus the contribution of original features in classification is not easily interpretable. These are two major drawbacks of this method which highlights the need for other dimensionality reduction methods [59].

#### 2.3.7. Feature Selection

As mentioned in the previous part, some extracted features are not necessarily informative in model creation. Redundant or irrelevant features decrease processing speed and mislead the algorithm [60]. A proper collection of most significant attributes can boost the classification performance. Feature selection mostly is done prior to application of machine learning in order to speed up the model training time, make a simpler, easily interpretable model and enhancing the generalization power of the model [61, 62].

There is a variety of feature selection techniques such as filter, wrapper, and embedded method. They rank the features based on their importance and then pick an appropriate subset of features based on different approaches.

Filter method selects variables regardless of the model and only evaluates the intrinsic importance of the individual features. It ignores potential interactions between the subsets' elements and suppresses the least interesting variables. This method is effective in computation time and is robust to overfitting. Overfitting is a modeling error which occurs when a function is too closely fit to the training data points so this complex model fails to perform well in the training set. However this technique does not consider the relationships between variables so it tends to select redundant variables [63]. In contrast to filter method, Wrapper considers the probable interaction between subsets' feature but it also has the risk of overfitting and it takes significant computation time to complete [64]. Embedded method is a combination of the both aforementioned methods. This algorithm takes advantage of its own variable selection process and performs feature selection and classification repeatedly until it reaches the best performance [65].

The authors of [66] sought to rank the contribution of features in the classification of the Axonal Projections neurons. For this purpose, they repeat the classification process multiple times and leave one feature out in each run. The amount of error growth demonstrates the importance of the leave out feature. Repeating this technique for each feature, all the features were ranked based on their importance.

To consider the feature interactions and correlations, Sun et al. demonstrated the performance of their novel feature selection algorithms on the neurons' morph repository. In their Binary Matrix Shuffling Filter (BMSF) algorithm, a matrix with the same column size of the original feature set and a subset of rows (samples set) are selected. Cells in each row are randomly assigned zero and one, representing absence or presence of that feature, where the total number of zero and one is equal to each row. Obtaining a reduced training set with this shuffling technique, the accuracy of SVM on the selected subset is measured via tenfold cross validation. Classification accuracy is calculated multiple times in the shuffled subset. Each time one column cell content is triggered while remaining part of the matrix left untouched to see whether that feature improves or degrades the accuracy. In former case, the feature will be kept in the final selected subset; otherwise it will be excluded. This step will be repeated until no more change happens in the final subset. They applied the algorithm coupled with Support Vector Machine (SVM), Back Propagation Neural Network, and Naive Bayes and reported the highest performance of their proposed feature selection technique [67].

### 2.4. Unsupervised Learning

Defining the hidden structure of the data, without any prior knowledge, is called unsupervised learning. A great amount of unlabeled data is fed to the algorithm and clustered data is achieved as an output. Clustering is the most popular unsupervised learning method which has been widely used in discrimination of unlabeled data so far. The majority of published research in automatic neuron classification has applied Ward's method, K-means, affinity propagation clustering, or a combination of those. Several samples are briefly reviewed in this section.

One of the conventional and widespread clustering methods in neuron classification is Ward's method. Main properties of the algorithm which make it popular are listed here: (1) most of the members of a group have common features, (2) each feature is visible in a large number of members, and (3) there is no need for all the members to have all the features [68]. This algorithm has the bottom-up strategy which means it starts from the leaves, groups close features together based on the overall largest similarity, and then makes a new cluster. It gradually follows this grouping technique in different levels of the tree until reaching a common root at top of the tree.

Tsiola et al. after applying PCA employed Ward with Euclidean metric for distance measurements on their own prepared dataset of 158 images of primary visual cortex neurons in mouse. They focused on dendrite and somatic shape. Five classes of cell emerged, including large pyramidal neurons, polarized nonpyramidal neurons, and short pyramidal neurons [45].

Despite the popularity of this approach among many neuroscientists, Ward's method has a major drawback. There is no chance for changing a data point which is assigned to a cluster after going up in the tree and recognizing a better cluster for that point [43, 44, 56, 69–76].

Researchers have exploited the K-means algorithm to address the aforementioned deficiency of Ward's method. Comparing their result with Ward, they proved that K-means has superiority in neurons discrimination.

K-means clustering has a reverse approach in comparison to Ward which performs top down. The number of desired clusters is defined in advance and the algorithm dynamically corrects the assignment of neurons to the different groups by calculating the inner similarity of the members [77, 78].

Badea et al. separated adult mouse retina cells based on the multidimensional feature space using K-means. They applied Ward as well to compare the results. Authors coupled molecular composition and physiological properties; for example, they linked receptive field size and connectivity to the ON and OFF pathways of the neurons with the morphological features like arbor area and stratification, arbor area and stratification within the inner plexiform layer, branching, density, and radiality of the dendrites to make a diverse set of features. One of the disadvantages of their research, as they also explicitly mentioned, is that, by considering a conservative cut-off for defining the number of clusters, they may suppress some crucial clusters [79]

The main concern of the authors who used Ward and K-means is that all the features have the same rate and importance in the classification process. This application neglects the fact that some features in clustering are more important than others. For example, the stratification level in the IPL has significant importance in comparison to other features, so considering it in the same way as other features may cause unfit clustering [79].

Other researchers like Kong et al. also used K-means algorithm as an appropriate tool for clustering. They explained the shape of a series of 219 retinal ganglion cells in the mouse. In contrast to same neuron types in the monkey, cat, and rabbit, mouse ganglion cells are less distinctive and pose a serious challenge for identifying subtypes [80]. Each 3D image from the neurons was mapped to 42 quantitative features. By eliminating the redundant and uninformative features at the first screening, 26 features were achieved. Then a correlation matrix for the feature sets was created to identify highly related features. Keeping this group of features leads to a high dimensional space with no extra information for classification. The authors avoided human intervention for weighting the features. After empirical experiment based on the correlation matrix and sinuhe analysis, three most significant features were produced which are branching density, stratification depth, and the extent of dendrites [3]. Chunwen et al. also used clustering coupled with PCA. The main difference of this method with similar experiments is that they used the extracted dataset from the Neuromorpho.org website and they defined a naming schema based on the morphologies of each type of neurons [81].

In contrast to most of the neurons, some neurons pertain to more than one type rather than having a strict membership to a particular group. Batagglia et al. referred to the former cell type as archetypal and to the latter cell type as atypical [82]. They proposed a fuzzy clustering algorithm to effectively identify the membership degree of atypical neurons to the main archetype. The fuzzy theory was invented by [83] in order to describe indefinite phenomena with a precise alphabet. Batagglia et al. used the same dataset as [43] and had the same approach; however, they focused on clustering the atypical cells. In their fuzzy clustering scheme, one neuron can belong to more than one class type with a different degree of membership. The sum of all membership degrees for a neuron in normalized form should be equal to 1. When one neuron is completely matched with one class, its membership degree is 1 and when it does not belong to a class, its degree is 0. In a study by Ristanovic et al. large sets of dentate nucleus morphology and topology were qualitatively and quantitatively analyzed. Seven features were extracted from the 2D images of neurons and were classified manually into four different groups. To verify findings and to show the consistency of the proposed classification schema, they applied T-test and ANOVA test [84].

Authors of [85] explored affinity propagation clustering on the dataset of 337 interneurons and compared it with the Ward algorithm. Results obviate a slightly better performance of affinity propagation in comparison to the Ward. The dataset was comprised of 20 electrophysiological and 67 morphological features. Considering only the shape of the neurons, 10 clusters appeared and by considering the physiological features, 36 clusters appeared. By combining all the features and applying the affinity propagation algorithm, 8 clusters with an accuracy of 77% were achieved.

A study that approaches the problem from a relatively dissimilar perspective has been done by DeFelipe, Lopez et al. [86, 87]. A taxonomic solution based on axonal arborization patterns was presented. Six axonal morphological features were defined to categorize GABAergic neurons which are less controversial cell types. After defining six features clearly, an interactive web-based system was created to allow 42 neuroscientists to ascribe the categories of the neurons in 320 images based on those features. The image repository was a collection of interneurons images of different parts of the cerebral cortex of human, monkey, cat, rabbit, rat, and mouse. A Bayesian network model was created to analyze different experts' answers. In order to ensure that the agreements were not accidental, Fleisss pi and Cohens kappa index were calculated. Eventually, an automatic clustering algorithm separated the dataset and output clusters corroborated the correctness of the community consensus.

There are multiple research findings for automatic classification and clustering of neurons based on other features (neuromorphological, electrophysiological, and molecular), which have used similar approaches [6, 70].

### 2.5. Supervised Learning

In spite of the sheer amount of labeled data, most of the researches in classification of the Neuromorpho space have used unsupervised machine learning techniques so far. However, these days public available databases like Neuromorpho.org proliferate the application of supervised methods. As it is often found that supervised techniques can perform comparatively better than unsupervised, this line of study progressed toward using labeled datasets [88].

Guerra et al. utilized supervised classification instead of unsupervised clustering in order to reap the benefits of prior knowledge in the field. They attempted to distinguish neocortical pyramidal cells from interneurons in a total of 327 samples. They compared the accuracy of Decision Tree, Naive Bayse, Multilayer Perception, Logistic Regression, and k-Nearest Neighbors algorithms with an unsupervised method. Additionally, they applied some dimensionality reduction techniques, like PCA, and feature subset selection to reduce the features number [88]. Their final dataset consists of 128 samples of pyramidal cells and 199 samples of interneurons from mouse neocortex with 64 extracted features and Apical Dendrites as the label set. A comparison of the outcomes proves the superiority of the supervised classification approach and the effectiveness of the dimensionality reduction and feature extraction methods in this specific morphological task.

After creating 400 pairs of image stacks from a pool of motor neurons in the Drosophila larvae and converting them to 2D images in the lab, Chang et al. partitioned neurons based on their three main morphological parts, soma, axon, and dendrite. Neurons were annotated manually into five separate subtypes. Chang et al. applied their proposed algorithm named “hNNRF-T: Hidden Neural Network Random Field” to classify the dataset. The input of the Neural Network is the morphology features from different neuron parts, the hidden layer is a sigmoid nonlinear function, and the output is the energy which controls the interactions in the hidden conditional random field. They tested Support Vector Machine (SVM) with Gaussian kernel, a Logistic Regression Model, and a Gaussian Mixture Model (GMM) on the dataset and compared outcomes to demonstrate the superiority of their model. The accuracy of the proposed method shows the higher performance of the hNNRF in the classification of their specific sample set of neurons. While the method of converting 3D images to 2D by preserving the whole content of the image is inspiring, there is not enough evidence to prove that this method outperforms all of the state-of-the-art algorithms in a more general dataset like Neuromorpho.org [89]. Zhao and Plaza have proposed a method in which electron microscopy images of drosophila optic medulla are fed into the segmentation part while labeled field output is the input of the skeletonization part. This skeletonization part converts the binary image to a skeletonized model, which is a 3D neuron-shaped using the TEASAR method. Different skeletons based on different inputs were provided and compared with a set of predefined skeletons. Features were then calculated. After a pairwise matching between different feature sets as a signature of each image, a similarity matrix was made. Then in the final step, they applied the affinity propagation clustering algorithm and K-Nearest Neighbor classification on the normalized similarity matrix. Different classes of the most similar neurons emerged as the desired result. The authors proposed their idea that the location of the branches determines the types of the neurons. They also tried to implement a detection algorithm based on the branch density. Although the accuracy of their method is high in the provided dataset, it is not comparable with other algorithms which have been applied in public dataset. The dataset was created manually in their laboratory and a specific alignment was needed to achieve an acceptable result, which was a major drawback of their proposed method [90].

Recently, Sun et al. exploited a Support Vector Machine (SVM) paired with their proposed method of Binary Matrix Shuffling Filters for Feature Selection (BMSF). BMSF is a feature selection technique (mentioned in the feature selection section) which is coupled with a classifier to define the neural space boundaries. They also coupled their BMSF methods with other state-of-the-art classification algorithms and compared the classification accuracy of those methods including Back Propagation Neural Network (BPNN), SVM recursive feature elimination (SVM-RFE), and Naive Bayes, with and without their proposed feature selection technique to prove the effectiveness of the proposed method. [67].

Jiang et al. classified the neurons' space based on their morphological features. Acquiring the neuron's images from neuromorpho.org and extracting 20 features per neuron's image, they applied PCA to reduce the feature space to only four features. By employing a back propagation algorithm, they classified the space into various subtypes which have different functionalities including Purkinje, motor pyramidal sensory neurons, and interneurons [91].

### 2.6. Multilabel and Multiclass Classification

In some of the classification problems multiple classes should be predicted rather than binary division of the space. Furthermore, not only classes are more than one but also there are more than one label for different classes. This kind of problems is considered multiclass multilabel classification. Neuron's morphology classification can fall in this category [92].

Fernandez et al. compared several state-of-the-art multilabel classifiers on the Neuromorpho.org dataset in order to detect gender, species (rat, human, mouse, and elephant), developmental stage, area of the neocortex (fronto-insula, anterior cingulate, motor, somatosensory, entorhinal, occipital lobe, frontopolar, multiple, frontal lobe, insular cortex, precentral gyri, postcentral gyri, and media prefrontal cortex perirhinal), cell type level one (interneuron or principal cell), and cell type level two (stella, pyramidal, basket or bitufted, neurogliaform, and containing cell). They formulated this multilabel classification problem and introduced Class Bridge decomposable Multidimensional Gaussian Classifier (CB-MGC). The model is a variation of the Bayesian network classifiers and outperforms all the state-of-the-art multiclass, multilabel algorithms. They reported their results with the performance measures like hamming score and exact match. Based on the aforementioned metrics, their proposed algorithm shows higher performance [93].

The presented experiments and research were some of the most significant efforts that have been done toward automatic classification of the neuron space. Although most of them apply the methods in a locally created dataset, the idea can be exploited and applied in a large enough public dataset to derive a more general conclusion about the performance and feasibility of the automatic classification.

## 3. Neuron's Retrieval Methods

The deluge of online neuron 3D images has led to an increasing demand for exploring, identifying, analyzing, and retrieving image data. This trend stimulated research seeking to develop an efficient high-speed retrieval algorithm for looking into datasets and satisfying neuroscientists questions and needs.

Considering this demand, Costa et al. implemented software named NBLAST. This software provides various functionalities to search and retrieves neuron images in a database and it has the capability of defining the well-matched type for a neuron based on its image. NBLAST is also capable of detecting two different images of the same neuron, and it can differentiate neurons of two highly similar types. NBLAST decompose the neurons' images into small segments, and by applying log-likelihood score matrices it defines the matches in the dataset. This algorithm makes a hierarchical clustering by applying Ward and affinity propagation method and groups the neurons' images based on the features similarity. It provides a ranked list of possible similar neurons which makes the search faster and more efficient [94]. However, assigning a huge feature vector to each image of a tremendous database leads to considerable response time.

While search speed is an important issue, a group of researchers aimed at tackling this problem by exploiting the hashing concept. In an image hashing search, each image is converted to a binary code which needs far less space to be kept in the memory and is easier to be explored. Although Weiss et al. proved that finding the best codewords is an NP-hard problem they proposed a novel formulation for redressing the issue, called Spectral Hashing (SH) [95]. Several other efficient encoding schemes were proposed to improve searching performance in big image databases such as Neuromorpho.org. Considering that neuron image repository is a giant dataset, researchers apply hashing concept to provide the fast searching capability [96, 97].

Traditional metrics like Euclidean distance for calculating the similarity between high volume of 3D images are inefficient so Li et al. implemented a new idea for rapidly searching and retrieval in large-scale 3D space of the neuronal databases. They proposed an efficient asymmetric binary coding function to implement a high-speed maximum inner product search (MIPS). This algorithm not only saves more space as a result of the compact representation of each image by 32 bits but also speeds up the search time among huge databases, like Neuromorpho.org, by an order of 30 times. They compared their algorithm with the different state-of-the-art algorithms, like Anchor Graph Hashing (AGH) [98], Spectral Hashing (SH) [95], and Iterative Quantization (ITQ) [99], to demonstrate its superiority. In brief, their proposed algorithm first extracts the images features and then applies the maximum inner product search to find the best binary coding function. After getting the best function, it converts each image into 32-bit binary code. At the retrieval time, it converts the query image to binary code with the same function and uses an inner product search to find the most similar images in the dataset. In their later work, they added two extra steps to improve the efficiency of the algorithm. After feature extraction, they grouped features into different hierarchies to create the similarity matrix. And also after maximum inner product search, an asymmetric optimization is applied and two coding functions are generated [100, 101].

Several retrieval techniques have been adopted for large-scale medical image analytics which also have the potential to be applied for mining the neuromorphological space. For instance, Zhang et al. represented the histopathological image data by various features such as image histograms, bag of words, local texture, and shape. They used these huge feature vectors to find similarities among different clinical cases. To improve search speed in the high dimensional feature space, they applied kernelized and supervised hashing methods as a scalable query method. They validated their method on the cell-level analysis of thousands of breast tissue images [102, 103]. Although the histopathological image data is relatively different from neuromorphological image data, the idea of applying kernel and changing the space can be applied for neuronal space. A summary of applied machine learning techniques in the neuron's morphology research is provided in Table 2.

## 4. Morph Is Not Enough

Neuroscientists recently agreed that neuron classification cannot be satisfying unless considering multimodal information of neurons rather than only morphological features [28]. Following this fact, Karagiannis et al. classified a group of neurons based on physiological, molecular, and morphological features. They selected the Neuropeptide Y (NPY) neurons which have three distinctive subtypes with different sets of properties. They extracted some morphological properties such as orientation of major axis relative to the radial axis and laminar location. They also took into account the electrophysiological and molecular properties such as first spike amplitude and NPY marker to have a comprehensive pool of neuron's features. The authors aimed at clustering the neurons based on the mentioned attributes into three main types: bipolar, tufted, and multipolar. They applied the Ward clustering technique and they demonstrate that more robust clustering schema is achievable by considering wide variety of neuron's features rather than only morphology [43]. Several other studies have classified neurons based on different combinations of morphological, physiological, and molecular properties using unsupervised clustering algorithms with a relatively similar approach [6, 56, 66, 70, 104–106]. All the aforementioned studies attempt to demonstrate that a comprehensive diverse feature set leads to a more accurate classification results.

## 5. Computational Methods Validation and Comparison

To this part, most of the distinguished studies and research in Neuromorpho space have been reviewed. Following the proposed pipeline, in this section, we try to classify the public Neuromorpho database and present the outcomes.

As the first step, we download a large portion of the neurons' images from different categories in the Neuronorpho.org site. A pool of neurons composed of 16647* drosophila*, 173 human, 1181 mice, 6426 rats, 184 monkeys, 300 giraffes, 302* C. elegans*, 360 chimpanzees, 127 crickets, 143 humpback whales, 95 elephants, and 60 minke whales samples are collected. Features of each image have been extracted with the help of L-measure. Afterward data has been cleaned, missing values were replaced by zero, and naming was standardized and preprocessed, and at the end a group of classifiers such as Random Forest, Decision Tree (J48), K-Nearest Neighbors, Logistic Regression, and Naive Bayse have been applied to classify the data. The accuracy of each algorithm considering different label sets is provided in the Table 3.

Well-matched with previous studies [107], Random Forest demonstrates an outstanding performance among other classifiers.

Random Forest is a type of supervised machine learning algorithm which is ensemble of multiple decision trees. For each tree in the forest a bootstrap sample of data is taken to create various input dataset so that each tree will be fit in a different set of samples. Then the data will be split based on a selection of random variable. The best split will iteratively be selected based on the impurity measure. The whole process will be repeated to build several decision trees to complete the Random Forest model. Each new data point will be fed iteratively into all the generated trees and their outcome result will be averaged to form the final prediction of the Random Forest. This algorithm achieves the highest accuracy of over 85% among other classifiers for predicting the developmental stage, species type, primary cell type, and gender. However, for predicting secondary and tertiary brain cells, the performance is still too low due to the great diversity in the subtypes and shortage of data in each subtype.

The rat, mouse, monkey, human, chimpanzee, and drosophila sample sets were also classified with the help of Random Forest classifiers and results are shown in Table 4.

## 6. Future Directions

Despite different approaches for neuromorphology presented in this work, there are other promising directions that should be explored and there are several issues that should be addressed to get more reliable results. Here we briefly mentioned the open challenges.

### 6.1. 3D Image Classification

The majority of the applied algorithms for classification of the neurons have been developed based on the extracted features by the software like L-measure, Cvapp, Neuronal, and Neurolucida Explorer [39]. To the best of our knowledge, direct processing of the 3D neurons' images has been never explored in this field. Applying convolutional neural network and deep learning techniques for directly processing and extracting the features of the reconstructed 3D image is a promising direction.

### 6.2. Enormous Database Retrieval

Although several hashing ideas have been applied in the neuronal space, still there are much more potentially effective methods that have not been examined in this space. Jiang et al. have exploited joint kernel supervised hashing method with multiple features for retrieval of the histopathological breast cancer images. To preserve the data similarity in the hamming distance, kernel functions were introduced. After feature extraction, they applied different kernels for individual features and defined a joint kernel function which linearly combines those kernels. Afterward a series of hashing function are constructed based on the joint kernel. A supervised algorithm is applied to optimize the weights and hashing functions, based on the provided images' metadata. Hashing function helps to reduce the high dimensionality of the feature space and makes a compact hash code which boosts the search speed [108]. This research idea has great potential to be applied in neuronal space.

### 6.3. Heterogeneity

Traditional neuromorphology research usually lacks standardization and often fragmented with multiple formats, prepared with different standards and various naming schema. Therefore a common data standard development is an absolute need for achieving more homogeneity and consistent results. Confounding variables should be defined and removed from the dataset. In addition, there are problems of missing values and noise issues that should be addressed prior to the huge datasets classification.

### 6.4. Generalization

Most of the aforementioned studies focused on a locally created dataset. Some of them only studied a part of a public dataset. They applied and tested their proposed algorithms and methods on a limited incomplete data and have provided the results independently. These separate efforts and research, while they are valuable but lack the generality to induce a fact or reach a conclusion based on them. A consistent study on a comprehensive dataset is needed to obviate the semantic dependencies and hidden rules in a more generalized and complete form. Finding a high performance and accurate model for classification of neurons will help to complete the chain of automatic neurons type detection.

## 7. Conclusion

This article presents a comprehensive overview of the techniques, pipeline, future directions, and challenges for neuromorphology in the big data age, by presenting a structured analysis of the neuromorphology methods in nearly 100 papers and web articles. We have summarized most of the important machine learning techniques that have been used for this purpose so far and we have provided a systematic data processing pipeline for the automatic neuron morphology. Automatic classification of the neuron images in the big data age with the growing speed of the reconstructed neurons images is an emerging and highly important research field with potentially significant impact on the neurological disorders diagnostic. The future of this field will benefit from the exponentially increasing amount of digital neuron image.

## Figures and Tables

**Table 1:** A short list of tracing software and toolkits.

**Software/Toolkit**	**Web Address **	**Availability**	**Reference**
Neurolucida	http://www.mbfbioscience.com	Commercial	Halavi et al. [16]
NeuronJ^*∗*^	https://imagej.net/NeuronJ	Open Source	Meijering [17]
Simple Neurite Tracer^*∗*^	https://imagej.net/Simple_Neurite_Tracer	Open Source	Longair et al. [18]
Sholl Analysis^*∗*^	https://imagej.net/Sholl_Analysis	Open Source	Ferreira et al. [19]
NeuronStudio	http://research.mssm.edu/cnic	Open Source	Rodriguez et al. [20]
Vaa3D	http://www.alleninstitute.org/what-we-do/brain-science/research/products-tools/vaa3d/	Commercial	Peng et al. [21]
FARSIGHT	http://farsight-toolkit.org	Open Source	Luisi et al. [22]
NeuronCyto	http://neuroncyto.bii.a-star.edu.sg/	Open Source	Yu et al [23]
Aivia	https://www.drvtechnologies.com	Commercial	N/A
Imaris	http://www.bitplane.com/imaris-for-neuroscientists	Commercial	N/A

^*∗*^ImageJ plugin.

**Table 2:** Machine learning techniques for neuron classification.

**ML Technique **	** Algorithm **
Unsupervised Techniques	Ward
	K-Mean
	PCA
	Affinity Propagation
	Fuzzy Set Clustering

Supervised Techniques	Feature Selection
	Neural Network
	Hidden Neural Network Random Field
	SVM + Binary Matrix Shuffling Filters
	Multiclass Classification

Retrieval Techniques	Ward + Affinity Propagation
	Binary Hashing Search
	Maximum Inner Product

**Table 3:** Baseline accuracy of different algorithms based on different classes.

**Machine Learning Alg**	** Random Forest **	** Decision Tree **	**KNN **	**Linear Reg **	** Naive Bayes **
Species	**98.19%**	96.65%	93.6%	90.42%	78.2
Gender	**85.12%**	81.11%	82.57%	80.19%	78.2
Primary Cell Type	**86.08%**	83.67%	79.24%	73.44%	71.7
Primary Brain region	**68.43%**	61.21%	56.29%	48.07%	24.69
Development	**97.47%**	96.53%	94.89%	91%	83.08

**Table 4:** Random forest accuracy based on the different species.

**Species Name**	** Rat **	** Mouse **	** Monkey **	** Human **	** Chimpanzee**	** Drosophila**
Development	96.2%	97.3%	92.9%	100%	77.8%	99%
Gender	99.3%	99.4%	94%	91.3%	80.8%	79.1%
Primary Cell Type	97.7%	99.3%	98.4%	98.3%	99.8%	83%
Primary Brain Region	97%	96.9%	missing	98.8%	1	59%

## Data Availability

The public datasets of neuromorphology is available at http://neuromorpho.org/. Source code (Python and R scripts) implementing the methods and the analyses described in this paper can be requested from Maryamossadat Aghili at mailto:maghi001@fiu.edumaghi001@fiu.edu.
